# The role of integrins in TGFβ activation in the tumour stroma

**DOI:** 10.1007/s00441-016-2474-y

**Published:** 2016-08-12

**Authors:** Zareen Khan, John F. Marshall

**Affiliations:** Centre for Tumour Biology, Barts Cancer Institute, Queen Mary University of London, London, UK

**Keywords:** TGFβ, Integrin, αvβ1, αvβ3, αvβ5, αvβ6, αvβ8, Tumour stroma, Tumour microenvironment

## Abstract

TGFβ1 is the most pleiotropic of all known cytokines and thus, to avoid uncontrolled TGFβ-activated processes, its activity is tightly regulated. Studies in fibrosis have led to the discovery that αv integrins are the major regulators of the local activation of latent TGFβ in our tissues. Since all cells can express one or more types of αv integrins, this raises the possibility that, in the complex milieu of a developing cancer, multiple cell types including both cancer cells and stromal cells activate TGFβ. In normal tissues, TGFβ1 is a tumour suppressor through its ability to suppress epithelial cell division, whereas in cancer, in which tumour cells develop genetic escape mechanisms to become resistant to TGFβ growth suppression, TGFβ signalling creates a tumour-permissive environment by activating fibroblast-to-myofibroblast transition, by promoting angiogenesis, by suppressing immune cell populations and by promoting the secretion of both matrix proteins and proteases. In addition, TGFβ drives epithelial-to-mesenchymal transition (EMT) increasing the potential for metastasis. Since αv integrins activate TGFβ, they almost certainly drive TGFβ-dependent cancer progression. In this review, we discuss the data that are helping to develop this hypothesis and describe the evidence that αv integrins regulate the TGFβ promotion of cancer.

**Graphical Abstract:**
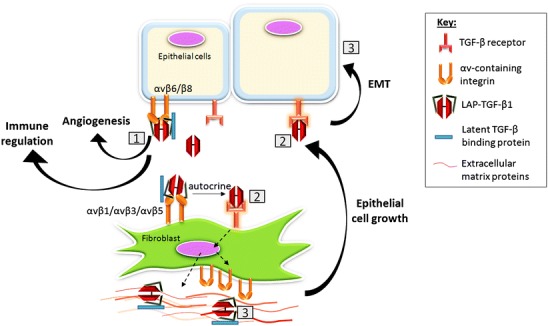
Mechanisms of integrin-mediated transforming growth factor beta (*TGFβ*) activation and its effect on stromal processes. *1* Matrix-bound latent LAP-TGFβ1 binds αv integrins expressed by epithelial cells or fibroblasts (*LAP* latency-associated peptide). TGFβ1 becomes exposed. *2* Active TGFβ1 binds the TGFβ receptor in an autocrine or paracrine fashion. *3* TGFβ1 signalling increases integrin expression, LAP-TGFβ1 secretion and trans-differentiation of fibroblasts into contractile cells that secrete collagens and collagen cross-linking proteins. By contracting the matrix, latent TGFβ1 is stretched making the activation of latent TGFβ1 easier and creating a continuous cycle of TGFβ1 signalling. TGFβ1 promotes cancer progression by promoting angiogenesis, immune suppression and epithelial-to-mesenchymal transition (*EMT*)

Mechanisms of integrin-mediated transforming growth factor beta (*TGFβ*) activation and its effect on stromal processes. *1* Matrix-bound latent LAP-TGFβ1 binds αv integrins expressed by epithelial cells or fibroblasts (*LAP* latency-associated peptide). TGFβ1 becomes exposed. *2* Active TGFβ1 binds the TGFβ receptor in an autocrine or paracrine fashion. *3* TGFβ1 signalling increases integrin expression, LAP-TGFβ1 secretion and trans-differentiation of fibroblasts into contractile cells that secrete collagens and collagen cross-linking proteins. By contracting the matrix, latent TGFβ1 is stretched making the activation of latent TGFβ1 easier and creating a continuous cycle of TGFβ1 signalling. TGFβ1 promotes cancer progression by promoting angiogenesis, immune suppression and epithelial-to-mesenchymal transition (*EMT*)

## Introduction

Transforming growth factor β1 (TGFβ1) is a pleiotropic cytokine that can function as both tumour suppressor and tumour promoter (for reviews, see Inman 2011; Yang and Moses 2008; Roberts and Wakefield 2003). TGFβ1-dependent tumour suppression is attributable mostly to the direct effects of the inhibition of epithelial cell division by the activation of cyclin-dependent kinase inhibitors (p21, p15 Ink4b). Once tumour cells develop mutations that render them refractile to the growth-suppressive effects of TGFβ1, then the cytokine can become tumour-promoting by acting directly on the tumour cells to drive an invasive programme but also indirectly by promoting a tumour-permissive microenvironment (Inman 2011).

In the last 20 years, it has become clear that the αv family of RGD-binding integrins are major regulators of TGFβ1-dependent processes in both normal and pathological processes (for reviews, see Wipff and Hinz 2008; Margadant and Sonnenberg 2010; Sheppard 2015). Some of these same integrins have also been strongly implicated in the promotion of the growth and spread of many different types of cancer. Therefore, integrins that are able to activate TGFβ1 in vivo are probably also likely to modulate tumour progression indirectly via the local production of active TGFβ1. Strong data also describe the way that TGFβ1 regulates the tumour microenvironment (TME) to regulate cancer progression. In this review, we will combine those data and extrapolate from a combination of in vitro and clinical observations to infer the likely roles of integrin-dependent TGFβ1 regulation of the tumour stroma. First, we will review briefly the integrin-dependent activation of latent-TGFβ1 and those integrins that are candidates for the activation of TGFβ1 in cancer.

## TGFβ is deposited in extracellular matrix as an inactive latent complex

The TGFβ family of cytokines and receptors includes activins, bone morphogenetic proteins and the TGFβ cytokines. In this review, we will concentrate on TGFβ1 but direct readers to a recent excellent review of other members of the family (Wakefield and Hill 2013). Three isoforms of TGFβ (TGFβ1, TGFβ2 and TGFβ3) are widely expressed in tissues but have different functions. Knockout of the TGFβ1 gene in mice (*tgfb1*) results in the infiltration of tissues with mononuclear cells eventually killing the animal within weeks of birth (Shull et al. 1992; Kulkarni et al. 1993), whereas the knockout of TGFβ2 (Sanford et al. 1997) or TGFβ3 (Kaartinen et al. 1995) causes developmental defects. All three isoforms of TGFβ bind to the same cell surface receptor TGFBRII, which exists as a dimer, a constitutively active serine-threonine kinase that, upon binding a ligand, dimerises with and phosphorylates a dimer of TGFBRI (also known as ALK5) forming a quaternary complex (Kang et al. 2009). This complex recruits and activates via phosphorylation the SMAD2 and SMAD3 transcription factors, which now can bind SMAD4 promoting their translocation to the nucleus in which the complex drives the transcription of a wide variety of genes, including the αv integrins (Levy and Hill 2005; Margadant and Sonnenberg 2010). TGFβ also activates multiple SMAD-independent signalling pathways that can promote tumour progression (Inman 2011).

TGFβ1 is a highly pleiotropic cytokine, which is probably the reason that its synthesis and activation is so tightly controlled. As described previously (Annes et al. 2004), the TGFβ1 gene product (TGFB1) is generated as a homo-dimer that is post-translationally cleaved by Furin-like enzymes into a larger pro-peptide, called the latency-associated peptide (LAP), which associates with the smaller TGFβ cytokine, the last-mentioned remaining non-covalently bound; this is referred to as the small latent complex (SLC). Two such processed proteins form a homo-dimer, which become covalently linked to one of four latent TGFβ-binding proteins (LTBP-1,-2,-3 and -4) within the Golgi prior to being secreted as a large latent complex (LLC) of LAP-TGFβ-LTBP. The LLC becomes anchored in the matrix via covalent linkage of LTBPs to fibrillin-1 (for a review, see Robertson et al. 2015). The concentration of TGFβ1 embedded in the extracellular matrix of normal organs has been estimated to be far in excess of that required to promote most TGFβ1-mediated processes (Sheppard 2015). However, while embedded within the LLC, the TGFβ1 remains inert. Thus, TGFβ1-dependent processes are controlled locally in the tissues by the regulation of the activation of latent-TGFβ1, which is, effectively, the exposure or release of the TGFβ1 cytokine to access by TGFβ1 receptors. This is important as uncontrolled TGFβ activity can lead to chronic fibrosis (Margadant and Sonnenberg 2010), which can be a significant risk factor for the development of some cancers (Hubbard et al. 2000; Bataller and Brenner 2005).

## Integrin activation of latent TGFβ in vivo

Munger and colleagues reported that the RGD-binding integrin αvβ6 activates latent TGFβ1 by binding to the RGD motif of LAPβ1 and exerting an actin-dependent physical deformation of the LLC, exposing the TGFβ cytokine to TGFβ receptors on adjacent cells (Munger et al. 1999). Of note, αvβ6 also binds TGFβ3 in a similar fashion. The process, which is protease-independent, has been subsequently shown to require fibronectin-anchored LTBP1 (Annes et al. 2004; Dallas et al. 2005). Biologically, the epithelial-specific αvβ6 is required to promote lung fibrosis in response to bleomycin (Munger et al. 1999) and, in separate studies, kidney fibrosis in response to deficiency in the collagen subunit Col4A3 (Hahm et al. 2007) and in both cases, antibodies to αvβ6 suppress fibrosis. Wipff et al. (2007) reported that thrombin-activated contraction by myofibroblasts activates TGFβ1 predominantly via αvβ5 and, less so, via αvβ3 or an unidentified β1 integrin (Wipff et al. 2007). The process of activation is also wholly mechanical, since detergent-treated cells lacking any cell membrane or cytosol can still activate latent TGFβ1 in response to exogenous ATP; again, αvβ5 blockade reduces this TGFβ1 activation by 66 %, twice as much as a blockade of αvβ3 or β1 integrins. Henderson et al. (2013) reported that mice globally deficient for integrin subunit genes, namely *itgb3*, *itgb5* or *itgb6* and selectively deficient for *itgb8* in haemopoietic cells are not protected from experimental liver fibrosis induced by carbon tetrachloride treatment, whereas mice with myofibroblasts deficient in the αv gene (*itgav*) are protected (Henderson et al. 2013). Using an αvβ1-specific peptide inhibitor, Henderson and colleagues confirmed that αvβ1 can promote liver fibrosis, possibly identifiying the TGFβ1-activating β1 integrin earlier described by Wipff et al. (2007). These data show that integrin-dependent TGFβ1 activation can vary between tissues and cell types. Henderson and colleagues also concluded that αvβ1 promotes bleomycin-induced lung fibrosis in mice. Since the same group has previously reported αvβ6 as being the driver of bleomycin-induced lung fibrosis (Munger et al. 1999), these results suggest that two or more TGFβ1-activating integrins can operate simultaneously in a single tissue to regulate the stromal response. This has implications for the TGFβ1-dependent modulation of the tumour microenvironment in cancer.

In contrast to other integrins, αvβ8 activates latent-TGFβ1 in a protease-dependent manner by the co-localisation of membrane-bound protease MT1-MMP/MMP14 (Mu et al. 2002). Thus, αvβ8 binds to LAPβ1 or LAPβ3 and brings the latent complexes into proximity of the membrane-bound protease that cleaves the LAP. Integrin αvβ3 might also be capable of using proteases to activate latent TGFβ in vivo, as reports exist that αvβ3 can localise activated matrix metalloproteinase 2 (MMP2) and MMP9 at the cell surface (Brooks et al. 1996; Rolli et al. 2003) and these MMPs have been implicated as activators of TGFβ in vitro (for a discussion, see Jenkins 2008).

The specific importance of RGD-binding integrins as regulators of TGFβ activity in vivo has been confirmed by the creation of mice with LAP but containing the non-integrin binding motif RGE, instead of RGD. These mice have a phenotype that mirrors that of TGFβ1-deficient mice (Yang et al. 2007). However, despite the evidence that all αv integrins appear to activate latent TGFβ, mice deficient in αvβ3 or αvβ5 do not exhibit TGFβ1-deficient phenotypes, whereas mice deficient in αvβ6 exhibit skin and lung inflammation (Munger et al. 1999), the spontaneous development of several types of cancers (Ludlow et al. 2005) and emphysema in older mice (Morris et al. 2003), all phenotypes linked to deficient local TGFβ1 activity. Similarly, whereas the complete ablation of integrin αvβ8 is a lethal phenotype (Zhu et al. 2002), loss in dendritic cells results in chronic inflammation and autoimmunity, almost certainly attributable to the inability of dendritic cells to activate TGFβ1 and thereby regulate the production of TReg cells (Travis et al. 2007). Thus, the apparent ranking in the ability of integrins to activate TGFβ1 in vivo might be linked to their affinity for LAP. The sequence RGDLXXI, as occurs in LAPβ1 and LAPβ3, forms two binding sites for αvβ6: the RGD loop and an adjacent helix that presents a hydrophobic leucine/isoleucine-binding site permitting high affinity binding (DiCara et al. 2007) and probably also explaining the high affinity of αvβ8 for LAPβ1 (Mu et al. 2002). In contrast, no reports are available for a second binding site in LAPβ1 for other αv integrins. However, if the threshold for activating TGFβ1 was lowered, then the weaker binding αv integrins, namely αvβ5, αvβ3 and αvβ1, might play a larger role in local TGFβ1 activation. As discussed below, this is likely to occur.

As mentioned above, researchers at the Hinz laboratory reported that myofibroblasts express several αv integrins that can activate TGFβ1, including αvβ3, αvβ5 and an unidentified β1 integrin. They found that the integrin activation of TGFβ1 does not occur on soft pliable substrates but also established that the stretching of adherent myofibroblasts is sufficient to increase their ability to activate latent TGFβ1 (Wipff et al. 2007). More recently, Klingberg et al. (2014) showed that, when myofibroblasts remodel the extracellular matrix through their contractile activity, they also organise LTBP1 into fibrillar fibronectin deposits, whereas normal fibroblasts are less able to achieve this (Klingberg et al. 2014). Furthermore, myofibroblasts can activate twice as much TGFβ1 on the myofibroblast-organised matrix than the fibroblast-organised matrix, suggesting that placing the matrix under tension primes the latent TGFβ1 for activation. This has been established by using a strain machine that stretches cell-depleted extracellular matrices before cells are added. Myofibroblasts activate TGFβ1 more efficiently on stretched matrices and this correlates with the stretching of the LTBP1-Fn deposits, as observed by fluorescence microscopy. Indeed, a fraction of TGFβ1 is activated independently of integrins if the matrix is sufficiently stretched.

**Fig. 1:**
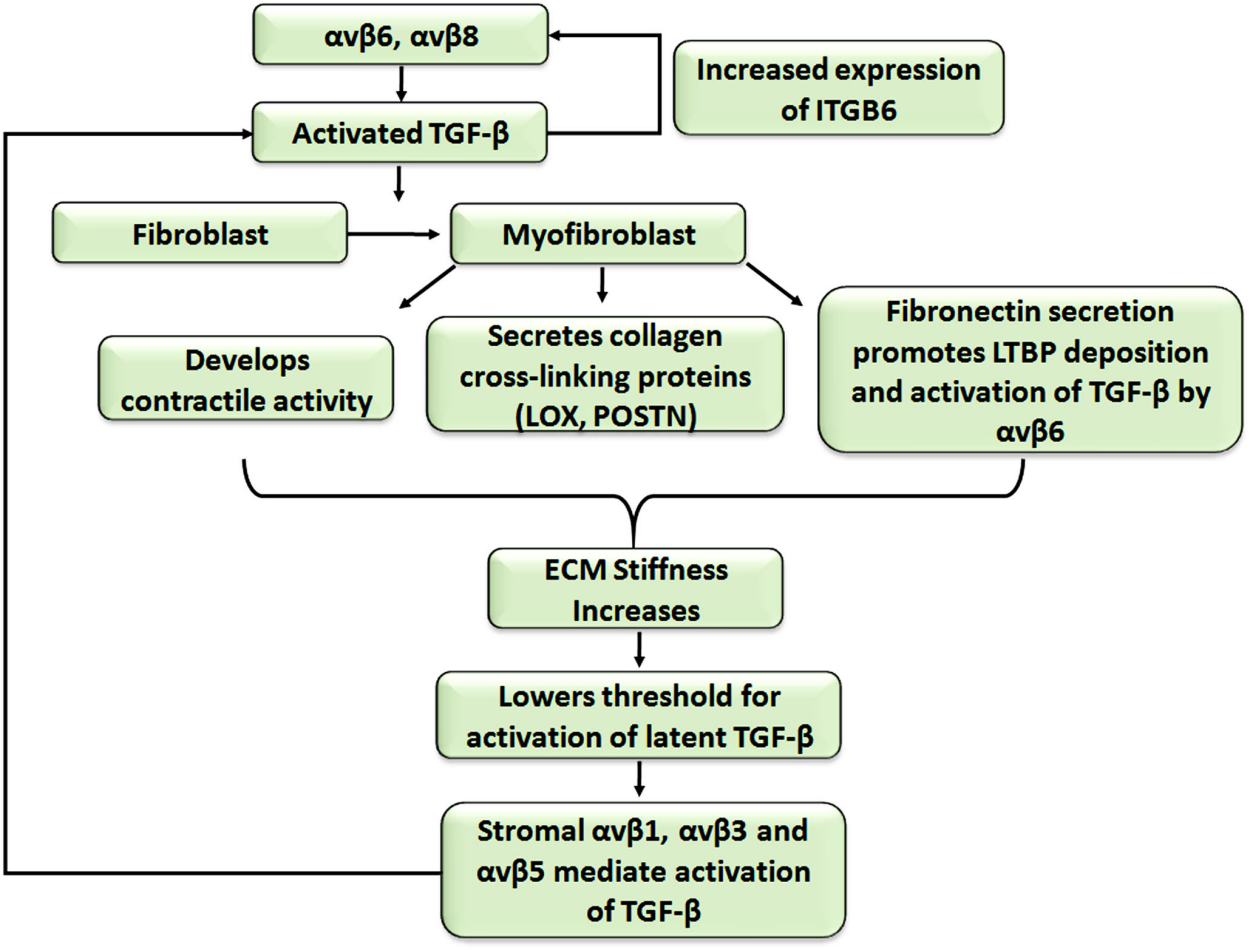
Cycle of contribution of transforming growth factor β1 (*TGFβ1*)-activated fibroblasts to extracellular matrix (*ECM*) composition and stiffness and further TGFβ1 activation (*LTBP* latent TGFβ-binding proteins, *ITGB6* integrin subunit gene 6, *LOX* lysyl oxidase, *POSTN* periostin)

Thus, non-proteolytic TGFβ1 activation by αv integrins is regulated at many levels: (1) sufficient amounts of an appropriate LAP-binding integrin must be present, (2) the integrin must be in the ligand-binding activated state (Wipff et al. 2007), (3) the cytoplasmic tail must be linked directly to the actomyosin cytoskeleton, (4) the extracellular domain is bound to LAPβ1 or LAPβ3 of the LLC, (5) the LLC must be anchored to a strain-resistant matrix in order to permit actin-dependent stretching of the complex to reveal the TGFβ. Finally, if the responding cell is adjacent to these activating events, this maximises the controlled local activation (Munger et al. 1999). Such tight control allows the exquisite regulation of TGFβ1 in our tissues. However, once the process has started, the activation of latent TGFβ1 might be rapidly amplified through integrin-dependent positive feedback loops; this is likely to happen in both fibrosis and cancer. Thus, as described in Fig. 1, the initial activation of TGFβ1 can increase the expression of more TGFβ-activating integrin subunits on both epithelial cells and local fibroblasts through SMAD-dependent signalling (Margadant and Sonnenberg 2010). Moreover, the activated TGFβ1 activates local fibroblasts to become myofibroblasts (discussed below), which develop contractile abilities and contract the extracellular matrix. The contracted matrix stretches the embedded LLC and lowers the threshold for activating more latent TGFβ1, potentially allowing integrins with a lower affinity for LAP (αvβ5, αvβ1, αvβ3) to activate more TGFβ1. The additional TGFβ1 generated further amplifies the process potentially resulting in a cycle of TGFβ1 activation-fibroblast activation-extracellular matrix stiffening-TGFβ1 activation etc. Thus, cancers can progress to a point at which the tumour:stroma interaction develops into an activation loop whereby the upregulated αv integrins, increased matrix stiffness and probably proteases released by both tumour and myofibroblasts result in uncontrolled TGFβ1 activation. This raises the question as to which point(s) within this escalation of TGFβ1 activation therapeutic intervention is still likely to be effective. Logically, the early blockade of a limited number of αv integrins as shown in fibrosis studies (Munger et al. 1999; Hahm et al. 2007) will probably suppress TGFβ1 activation in the tumour microenvironment but, once escalated, TGFβ1 activation might occur via many mechanisms and, thus, multiple interventions will be needed, which will be therapeutically challenging. Notably, whereas the remaining RGD-binding integrins (α5β1, α8β1 and αIIbβ3) have not been reported to activate latent TGFβ1 directly, α5β1 is required for the αvβ6-dependent activation of latent TGFβ1 (Fontana et al. 2005) presumably by binding to the fibronectin trapping the LTBP1.

## TGFβ1-driven changes in the tumour microenvironment can promote cancer progression

Recent reviews have described the role of TGFβ1 in the regulation of the tumour microenvironment (Pickup et al. 2013; Lin and Zhao 2015; Guo et al. 2016). In this review, we will concentrate on the available evidence linking integrin-dependent TGFβ1 activation to many of these microenvironmental changes.

1. **Integrin activation of TGFβ drives epithelial-to-mesenchymal transition**

The epithelial-to-mesenchymal transition (EMT) is the process by which epithelial cells reduce the expression or function of the proteins that promote cell-cell and cell-basement-membrane adhesion and thus transition towards a more motile and sometimes mesenchymal-like phenotype. This transition is essential during stages of embryogenesis and wound healing but also occurs in pathological processes, possibly for the initiation of metastasis (Mamuya and Duncan 2012). TGFβ1 induces EMT by generating a transcriptional repression of the epithelial gene signatures, including cell-cell adhesion molecule E-cadherin but by elevating mesenchymal genes, such as N-cadherin, αSMA and vimentin in a SMAD-dependent and -independent manner, in part via activation of the transcription factors Snail and Slug (Naber et al. 2013; Medici et al. 2006). Thus, the integrin-dependent activation of TGFβ1 might be expected to promote EMT, as has been reported.

Bates and colleagues (2005) reported that high levels of αvβ6 in colon cancer reduced overall survival from a median of 16.5 months in the αvβ6-low/negative cancers to 5 months in the αvβ6 strongly positive cancers. They also suggested a model of αvβ6-dependent activation of TGFβ1 and subsequent EMT as the potential mechanism for αvβ6-driven colon cancer (Bates et al. 2005).

Workers at the Danen laboratory discovered that the reduction of β1 integrin expression in breast cancer cells suppressed tumour growth but enhanced metastasis to the lungs. In three-dimensional (3D) matrices in vitro, similar treatments with integrin β1 inhibitory antibodies or genetic suppression of β1 expression resulted in a change from cohesive migration to single cell migration; this was dependent upon increased TGFβ1 signalling and was correlated with the loss of E-cadherin. Suppression of TGFβ1 signalling or increasing E-cadherin restored cohesive migration in β1-deficient cells and reduced their metastasis to the lungs (Truong et al. 2014). These observations were in agreement with earlier studies by Giampieri et al. (2009) who reported that activation of TGFβ1 signalling in a tumour cell population promoted a single cell migratory phenotype as opposed to cohesive cell migration in the absence of TGFβ1 signalling (Giampieri et al. 2009). These data link αv integrin activation of latent TGFβ1 directly to the metastatic propensity of cancer (Mamuya and Duncan 2012).

Sometimes, the upregulation of TGFβ1 signalling by integrins is indirect. Mori and colleagues showed that αvβ3 enhanced TGFβ1-induced EMT in MCF10A breast cells by binding directly to fibroblast growth factor 1 (FGF1) and making it available for binding to FGFR1 receptors. This FGF1-αvβ3 engagement and the FGFR1 signalling were necessary for FGF1 to maximise the TGFβ1-dependent EMT (Mori et al. 2015). TGFβ1 also elevates the expression of secreted matricellular glycoprotein fibulin-5, which contains an RGD-motif and is capable of binding α5β1, αvβ3 and αvβ5 (Yanagisawa et al. 2009). Fibulin-5 is expressed during development and is only re-expressed in injured tissues and cells undergoing EMT. Moreover, fibulin-5 stimulates MMP-2 and -9 expression and activity and promotes mammary epithelial cell invasion probably through an enhancement of Twist and reduction in E-cadherin expression. However, the direct link between fibulin-5 and integrins during EMT needs to be further established (Lee et al. 2008).

2) **Empirical and inferred data linking integrin activation of TGF to modulation of the tumour stroma**

We only recently established the central importance of the many different αv integrins in the activation of latent TGFβ and determined which αv integrins are likely to act when and where (see above). Therefore, relatively few studies have included integrin-blocking antibodies in their in vivo investigations in order to empirically determine which integrin, if any, is responsible for activating TGFβ1 and therefore is responsible for the TGFβ1-dependent effects on tumour cell growth and spread and the tumour microenvironment. In this section, we summarise both empirical data, whereby researchers have directly examined the role of integrin-dependent TGFβ activity in tumour progression and inferred data, whereby we can reasonably assume that integrin-dependent TGFβ1 activation is at play.

Van Aarsen et al. (2008) showed that Detroit-562 oral squamous cell carcinoma (SCC) xenografts developed a strongly positive αvβ6 invasive front and grew into a stroma rich in TGFβ1. Moreover, the xenografts exhibited a reticular αSMA pattern in the stroma at the periphery of the tumour correlating with the expression of αvβ6 and suggesting a relationship between the two proteins (Van Aarsen et al. 2008). A similar accumulation of αSMA-positive myofibroblasts abutting the invasive front of oral cancer, most of which were αvβ6-positive (Van Aarsen et al. 2008; Marsh et al. 2011), had been observed previously (Lewis et al. 2004). Treatment of Detroit-562 xenografts by systemic αvβ6-blocking antibody 6.3G9 or soluble recombinant TGFBRII-Fc protein suppressed tumour growth but did not significantly change the expression of αSMA, CD31-positive blood vessels or collagen deposition detected by Sirius red staining (Van Aarsen et al. 2008). In contrast, Eberlein and colleagues (2013) reported that the treatment of mice bearing Detroit-562 xenografts with 264RAD, a human antibody that inhibits αvβ6, resulted in a dose-dependent reduction in tumour growth that was mirrored by a dose-dependent reduction in fibronectin and SMA, two products of TGFβ-activated fibroblasts, suggesting strongly that αvβ6 was promoting a myofibroblast-rich microenvironment. Why these similar studies resulted in different apparent stromal effects remains unknown. Whether the small amount of αvβ8 blocking activity of the 264RAD antibody had any effect was not explored.

Moore and colleagues reported that αvβ6 co-operated with HER2 to promote HER2-driven invasive breast cancer (Moore et al. 2014). Treatment of mice with 264RAD suppressed the growth of both HER2-over-expressing BT474 and MCF7/HER2-18 tumours, an effect enhanced by combination therapy with the anti-HER2 antibody Trastuzumab. Analysis of tumours harvested mid-therapy showed that, in both tumour types, 264RAD therapy alone was sufficient to reduce total- and phospho-SMAD2 in the tumour and to reduce the number of αSMA-positive and endomucin-positive endothelial cells suggesting a direct link between αvβ6-dependent TGFβ1 signalling, the induction of myofibroblasts and the upregulation of angiogenesis.

Integrin activation of TGFβ1 influences cancer progression not only on the luminal component of breast tissue. Myoepithelial cells are the contractile cells adjacent to luminal breast cells that promote milk secretion from ducts. Allen et al. (2014) identified *de novo* expression of αvβ6 on breast myoepithelial cells as being a key biomarker associated with an invasive breast phenotype in ductal carcinoma in situ (DCIS; Allen et al. 2014). Moreover, a relationship existed between αvβ6-positive myoepithelial cells in DCIS and the subsequent recurrence of breast cancer. Since only 50 % of women with DCIS develop life-threatening invasive ductal carcinoma but still receive surgery and other therapies, this is an important observation with potential clinical ramifications. To determine the mechanism, Allen and colleagues showed that, when αvβ6 was over-expressed on normal myoepithelial cells, they could activate TGFβ1 to which they responded by upregulating MMP2 and MMP9. Blockade of TGFβ or MMPs inhibited the ability of αvβ6-expressing myoepithelial cells to promote breast cancer invasion. Analysis of DCIS samples confirmed a strong correlation of αvβ6 and MMP9 expression in myoepithelial cells. In further studies, the injection of αvβ6-positive myoepithelial cells together with MDA MB231 breast carcinoma cells resulted in a significantly faster growth of tumours compared with normal myoepithelial cells. Again, the data were consistent with the αvβ6 activation of TGFβ1 driving tumour cell invasion except that, this time, the αvβ6-positive “microenvironmental” myoepithelial cell promoted the invasive phenoptype.

Marsh and colleagues reported that, compared with the more indolent types of basal cell carcinoma (BCC), αvβ6 was selectively upregulated on the morphoeic type of BCC, a tumour that has a distinctly invasive and fibrotic phenotype (Marsh et al. 2008). As BCC is associated with the upregulation of Gli 1 and Gli 2 transcription factors, they were transfected into keratinocytes to generate an in vitro model of BCC. These cells were able to activate latent TGFβ1, which activated fibroblasts to become αSMA-positive myofibroblasts and which, in turn, secreted HGF that acted as a pro-invasive chemoattractant for the BCC cells. The data were consistent with increased αvβ6 expression by morphoeic BCC cells being significantly responsible for the invasive and fibrotic phenotype of this disease. Another skin carcinoma study also linked αvβ6 to the modulation of the TME. Patients lacking collagen 7 fibrils easily develop skin blisters that further develop into chronic fibrotic inflammatory lesions on bony joints and can often develop into invasive and metastatic squamous carcinomas (Fine and Hintner 2009). Martins et al. (2016) developed 3D tumour-mimetic organotypic gels in vitro combining a squamous cell carcinoma (SCC) cell line that had been engineered to lack collagen 7 gene (Col7A1) with fibroblasts in a collagen-based gel. Small-hairpin-RNA-treated SCC cells (shCol7 SCC) admixed with skin fibroblasts invaded significantly more than control cells and upregulated αvβ6 and the associated matrix exhibited a significant increase in fibronectin. The increased invasion, αvβ6 expression and fibronectin were all suppressed by co-incubation with a TGFβ1 receptor inhibitor. When the organotypic gels were implanted under the skin of mice to form tumours, again the loss of collagen 7 resulted in significant upregulation of αvβ6 expression specifically at the invasive front and a significant increase in fibronectin expression in the associated stroma and cancer. Moreover, increased phospho-Smad2/3 signalling was present in the stroma of the Col7-deificient SCC tumours. The authors suggested that Collagen 7 functioned as a TGFβ1 suppressor and, again, the data are consistent with an αvβ6-dependent activation of TGFβ1 activating a fibroblast-to-myofibroblasts transition, which subsequently increases the secretion of fibronectin.

Eberlein et al. (2015) investigated the effects of the co-culture of a panel of non-small cell lung cancer cell lines with fibroblasts and found that those cancer lines that promoted trans-differentiation of fibroblasts to myofibroblasts (αSMA–positive) expressed E-cadherin, EpCAM and αvβ6. Blockade of αvβ6 with 264RAD or of TGFBR1 with a small molecule inhibitor blocked myofibroblast formation. Interestingly, if the inhibitors were added 3 days after co-culture, only the TGFBR1 inhibitor suppressed SMA expression, suggesting that, whereas αvβ6 initiates the process, once started, αvβ6-independent processes maintain TGFβ activation (Eberlein et al. 2015).

Dutta et al. (2015) reported the novel αvβ6 regulation of TGFβ1 signalling in prostate cancer cells. Generating a panel of PC3 prostate cancer cells expressing αvβ6 or αvβ6/β3cyto or αvβ3/β6cyto chimeras, these authors reported that αvβ6 co-precipitated with TGFBRII, a relationship that required the cytoplasmic tail of β6 and that physical association was required for the TGFβ1-dependent upregulation of SMAD3, which in turn upregulated MMP2. This is the first report suggesting that αvβ6 regulates TGFβ1 signalling via physical association with the TGFβ1 receptor complex. The determination of whether this unique observation is seen in other cell models will be of interest.

Thus, numerous examples link the αvβ6-dependent activation of TGFβ1 as a promoter of pre-clinical tumour progression. Therefore, we must consider it likely that, in clinical studies in which poor overall survival has been linked to the over-expression of αvβ6, TGFβ1 activation by αvβ6 is a tumour-driver. Since Bates et al. (2005) reported that high αvβ6 correlates with poor survival in colon cancer, similar observations have been reported for non-small cell lung cancer (Elayadi et al. 2007), cervical cancer (Hazelbag et al. 2007) and breast cancer (Moore et al. 2014). Each of these studies had sufficient clinical samples and follow-up data to establish survival relationships. Other studies with smaller numbers of tissue samples also reported high fractions of carcinomas positive for αvβ6 including ovarian (100 %, Ahmed et al. 2002; 33 %, Van Aarsen et al. 2008), pancreatic (100 %, Sipos et al. 2004), oesophageal (68 %, Van Aarsen et al. 2008) and skin (84 %, Van Aarsen et al. 2008). Eventually, we will establish the role of αvβ6 and, indeed, the role of other αv integrins in activating TGFβ1 in each of these cancers and the subsequent effects on tumour growth and spread.

Notably, if cancer tissues retain the tumour-suppressive responsiveness to TGFβ1, then the blockade of αvβ6 could potentially promote cancer, as was described in a transgenic mouse model of pancreatic cancer (Hezel et al. 2012). In fact, early studies showed that, in normal tissues, αvβ6 was tumour-suppressive through its activation of TGFβ1. Thus, knockout of the β6 gene (*itgb6*) in mice was associated with a mild inflammatory response in skin and lung (Huang et al. 1996) and with a propensity for the spontaneous formation of certain cancers in up to 25 % of *itgb6*-null mice (Ludlow et al. 2005), patterns that mimicked a mild form of global TGFβ1 knockout mouse (Shull et al. 1992). These unrelated observations might have indicated that the αvβ6-dependent activation of TGFβ1 in part regulated tumour immune surveillance, as shown subsequently for αvβ8 (Travis et al. 2007). Certainly, a recent study reported that αvβ6 and αvβ8 on keratinocytes regulated the retention time of dendritic cells and memory T cells in the skin and colon by locally activating TGFβ1 (Mohammed et al. 2016).

Therefore, overall, whereas some data are conflicting, strong evidence has been provided that, in some cancer tissues, αvβ6 is associated with tumour promotion, in part via the activation of latent TGFβ1, which subsequently regulates elements in the stroma, including the number of myofibroblasts and blood vessels. In addition, some αv integrins clearly regulate the behaviour of inflammatory and immune cell populations. It is through these three processes, described below, that the αv integrin-dependent activation of TGFβ1 can impact most strongly on the stroma. Of note, the principal reason that most of the available data linking αv integrins to TGFβ-regulation of cancer involve αvβ6 is probably attributable to the absence of widely available reagents that specifically detect and inhibit integrins αvβ8 and αvβ1; this means their distribution and activity in cancer remains unknown. Moreover, whereas αvβ3 has been reported to regulate TGFβ1 signalling negatively in endothelial cells of healing wounds (Reynolds et al. 2005), the widespread expression of αvβ3 and αvβ5 on multiple cell types makes the determination of their specific activities difficult to prove by using global antibody blockade and will require genetic ablation in specific cell types.

3) **Myofibroblasts**/**CAFs promote cancer progression by multiple mechanisms**

TGFβ1 is the most powerful activator of the trans-differentiation of fibroblasts into myofibroblasts. Thus, those integrins that activate TGFβ1 can also induce myofibroblast formation and therefore can be considered responsible for myofibroblast-dependent activities that include matrix-remodelling, matrix-stiffening and cancer promotion.

All tissues have resident fibroblasts that maintain the tissue homeostasis of matrix content and whose presence primes tissues to respond to wounding or infection. However, TGFβ1 induces fibroblasts to adopt a contractile wound-repair phenotype and genotype termed a myofibrobast. When this trans-differentiation occurs in the tumour microenvironment, the myofibroblasts are also known as activated fibroblasts, cancer-associated fibroblasts (CAFs) or tumour-associated fibroblasts (TAFs). Not all CAFs are derived from resident cells. Thus, TGFβ1 can induce some non-fibroblast cells, including adipocytes (Jotzu et al. 2010) and circulating bone-marrow-derived suppressor cells (Weber et al. 2015), to trans-differentiate into cancer-associated myofibroblasts.

Integrins can also potentially regulate TGFβ signalling after TGF activation has occurred. In fibroblasts derived from human oral and dermal tissues, anti-αvβ3 or αvβ5 integrin antibody blockade in the presence of exogenous active TGF-β1 inhibits the expression of α-SMA and collagen gel contraction. Furthermore, in renal fibroblasts, only αvβ5 blockade and not αvβ3 is sufficient to reproduce these same inhibitory effects, suggesting that the differentiation of fibroblasts that are derived from different tissues are not necessarily regulated by the same integrin (Lewis et al. 2004). The mechanisms regarding the reasons that αvβ3 and αvβ5 are required for TGFβ1-induced trans-differentiation to the myofibroblast phenotype have not been established.

Above, we have discussed the way that, by increasing the contraction of the extracellular matrix, myofibroblasts increase the likelihood of activating latent TGFβ1. However, myofibroblasts secrete a huge number of proteins that further enhance cancer progression including extracellular matrix proteins, proteases, growth factors, cytokines and chemokines (for a review, see Miles and Sikes 2014).

Surgeons and pathologists have known for many years that most cancers are stiffer to the touch than the surrounding normal tissues and we now know that the cells responsible for this increased stiffness are the collagen-producing contractile myofibroblasts. Originally, the stiffness was assumed to be an effort on the part of the host to wall off the mutant tissue as a means of protection. Indeed, recent studies of pancreatic cancer suggest that some types of pancreatic fibroblast are tumour-suppressive (Rhim et al. 2014; Ozdemir et al. 2014). However, clearly, tumour cells often secrete latent TGFβ1 and can promote the generation of myofibroblasts and, thus, we can reasonably assume that they derive benefit from activating fibroblasts to myofibroblasts. We have only recently confirmed that a stiff extracellular matrix can signal tumour cells to promote their growth and survival (for a review, see Malik et al. 2015).

Myofibroblasts secrete many different types of collagens (discussed in Miles and Sikes 2014) that they form into fibrils in the matrix. A positive relationship exists between the thickness and orientation of the collagen fibrils and the invasive behaviour of cancers (Provenzano et al. 2006). Myofibroblasts can promote the cross linking of collagen fibres through the secretion of lysyl oxidases (LOX), which can covalently crosslink collagens and elastin. Many studies have reported that levels of LOX correlate with increased invasion, metastasis and poor survival in pancreatic cancer (Miller et al. 2015), astrocytoma (da Silva et al. 2015), breast cancer (Friesenhengst et al. 2014) and liver cancer (Zhu et al. 2015a). Such observations identify LOX and LOX-like enzymes as potential therapeutic targets.

Myofibroblasts also secrete periostin that acts in several ways to promote cancer. First, it can bind to fibronectin through its FAS domain and to bone morphogenetic protein-1 (BMP-1) through its EMI domain. This anchors BMP-1 in the matrix where it can promote the maturation of the pre-Lox propeptide into the mature Lox enzyme, enabling it to generate collagen crosslinks that can contribute to increase matrix stiffening (Maruhashi et al. 2010; Garnero 2012). Periostin is also a ligand for αv integrins. Periostin promotes the proliferation of melanoma cells via binding integrin αvβ3 and αvβ5 and activating the p43/p44 MAPK signalling pathway (Kotobuki et al. 2014). Moreover, the addition of TGFβ-neutralising antibody to cultures of fibroblasts in melanoma-cell-conditioned media prevents the upregulation of periostin (Kotobuki et al. 2014). Periostin also seems to drive oesophageal cancer. Thus, Underwood and colleagues (2015) reported that the number of αSMA-positive myofibroblastic CAFs in oesophageal carcinoma correlated with poor overall survival. Since up to 68 % of oesophageal cancers are reported to be αvβ6-positive (Van Aarsen et al. 2008), one could speculate that αvβ6 integrin induces these αSMA-positive cells via TGFβ1 activation. Moreover, the secretion of periostin by these oesophageal CAFs bind to αvβ3 and αvβ5 on oesophageal cancer cells activating PI3 kinase signalling and promoting oesophageal cancer cell invasion. Genetic ablation of the periostin gene (POSTN) in the CAFs eliminates the pro-invasive effects on the oesophageal cancer cells. Additionally, clinical material has shown an exact correlation between the expression of periostin and αSMA (Underwood et al. 2015). Given its dual role as a component of matrix stiffening and pro-invasive signalling, many recent reports have unsurprisingly linked periostin to cancer promotion and poor survival (Landre et al. 2016; Qin et al. 2016; Sung et al. 2016; Xu et al. 2016; Fukuda et al. 2015) suggesting that it is also a good potential therapeutic target for suppressing cancer development.

TGFβ1-treated fibroblasts can secrete osteopontin (Corallo et al. 2014), an RGD-containing integrin ligand implicated in the promotion of tumour growth, EMT and metastasis (Platzer et al. 2011; Kothari et al. 2016). Thus, increased osteopontin correlates with increased metastasis and often poor survival in multiple types of cancer including laryngeal squamous cell carcinoma (Chen et al. 2015), melanoma (Kiss et al. 2015), nasopharyngeal carcinoma (Hou et al. 2015) and breast cancer (Xu et al. 2015). Lenga et al. (2008) reported that osteopontin was required for TGFβ1 transdifferentiation of cardiac fibroblasts to myofibroblasts, whereas Weber et al. (2015) provided evidence that osteopontin induced bone-marrow-derived mesenchymal suppressor cells to become myofibroblasts.

TGFβ1 induces the transcription of the CCN family of matricellular proteins including CCN1/cysteine-rich protein 61 (CYR61) and CCN2/connective tissue growth factor (CTGF), which affect a variety of cell types, including fibroblasts, through binding to integrins (Leask 2013). CYR61 appears to function as a tumour suppressor or promoter depending on which integrin is engaged.The binding of CYR61 to α6β1 generates senescence-inducing reactive oxygen species in fibroblasts, associated with reduced TGFβ1 expression, or induces p53-dependent apoptosis, therefore protecting against aberrant cell proliferation and fibrosis (Jun and Lau 2010; Lau 2011). In contrast, the binding of CYR61 to αvβ3 or αvβ5 stimulates prostate cancer cell growth and metastasis (Grzeszkiewicz et al. 2001) or fibroblast migration (Sun et al. 2008). In oesophageal cancers, CYR61 mRNA is an independent poor prognostic factor (Huang et al. 2009).

CTGF promotes breast cancer EMT and increases collagen type I fibre deposition (Zhu et al. 2015b). In a separate study, the pro-fibrotic activity of CTGF was observed via a positive feedback loop, binding αvβ3 on fibroblasts and further stimulating αvβ3 upregulation, collagen synthesis and contraction, thereby enhancing the potential for increased TGFβ activation (Hu et al. 2014). In addition, intermediary proteins provide an extra level of regulation of TGFβ1 and integrin autocrine signalling. For example, TGFβ1 induces secretion of protease inhibitor plasminogen activator inhibitor-1 (PAI-1), which in turn can bind to and promote αvβ3 internalisation. Therefore, PAI-1 knockout in mouse embryonic fibroblasts results in reduced αvβ3 endocytosis, elevated transmembrane TGFβ type II receptor expression and enlarged focal adhesion sites, correlating with a three-fold rise in Smad2/3 expression (Pedroja et al. 2009).

Thus, these data and those of others (Beacham and Cukierman 2005; Sherman et al. 2014) suggest that a TGFβ1-activated myofibroblast/CAF-rich tumour stroma is tumour-promoting. However, conflicting data have also been presented. A recent study reported that the global depletion of replicating αSMA-positive myofibroblasts/CAFs in two transgenic mouse models of pancreatic ductal adenocarcinoma (PDAC) enhanced the development of undifferentiated pancreatic cancers. This was associated with a significant reduction in fibrosis, the down-regulation of infiltrating macrophages and the upregulation of immunosuppressive CD4+/FoxP3+ regulatory T cells (TRegs; Ozdemir et al. 2014). In separate studies, Rhim et al. (2014) examined the role of the sonic hedgehog signalling pathway, as sonic hedgehog (Shh) can promote a fibroblast-rich microenvironemnt in cancer. They showed that either genetic ablation of sonic hedgehog protein or pharmacological inhibition of the downstream smoothened protein resulted in the development of more aggressive, highly vascularised, PDAC tumours that had a significant reduction in stromal cell density (Rhim et al. 2014). In contrast, recent work demonstrated that TGFβ1 drove a pro-tumourigenic phenotype in pancreatic cancer (Principe et al. 2016). Using the Elastase-promoter driven KRas G12D mouse, which develops pancreatic neoplasia, Principe et al. (2016) eliminated epithelial TGFβ signalling selectively by over-expression of a dominant negative TGFBR2 (DN TGFBR2) construct or eradicated TGFβ1 signalling globally by generating *tgfbr1*+/− mice. Mice deficient in TGFβ signalling in their epithelia showed enhanced disease progression, consistent with the loss of TGFβ1-dependent tumour suppression. In contrast, the global knockout mice showed significantly reduced tumour progression, suggesting that TGFβ1 signalling in the stroma normally promoted pancreatic neoplasia. Whereas none of these recent studies included investigations of integrin expression, their contrasting observations concerning the stromal control of pancreatic fibrosis suggest that the genetic background of the transgenic models is a major determining factor for the outcome of such studies and that we still have much to discover.

4) **TGFβ1 enhanced angiogenesis**

Angiogenesis describes the formation of new blood vessels from pre-existing branches and promotes tumour progression, because blood vessels provide the essential nutrients required by growing tumours and facilitate metastasis (Chung and Ferrara 2011). TGFβ1 can promote angiogenesis (Vinals and Pouyssegur 2001; Li et al. 2001) and thus local activation of TGFβ by αv integrins in cancer is likely to promote the development of blood vessels. However, TGFβ-mediated effects are concentration-dependent, as low concentrations can promote blood vessel formation, whereas higher amounts are anti-angiogenic (Orlova et al. 2011). Moreover, TGFβ-concentration-dependent actions may occur via the stimulation of different TGFβ receptor signalling pathways. Activation of the activin-receptor-like kinase (ALK1) induces endothelial cell proliferation and migration via the phosphorylation of Smad 1/5, whereas the activation of the ALK5/Smad 2 cascade inhibits this effect on endothelial cells (Goumans et al. 2002).

Integrins αvβ3 and αvβ5 (Weis and Cheresh 2011) and αvβ8 (Zhu et al. 2002; Arnold et al. 2012) are all reported to regulate angiogenesis but only αvβ8 is described to do so through TGFβ1 activation. The role of αvβ3 and αvβ5 in angiogenesis is under debate. Both integrins are upregulated on endothelial cells of new blood vessels and promote their migration in vitro and their blockade with antibodies have suppressed blood vessel growth in a variety of pre-clinical studies (Gutheil et al. 2000; Friedlander et al. 1995). However, genetic ablation of *itgb3*, *itgb5* or both simultaneously accelerates tumour vasculature growth and development, attributable to the increased expression of vascular endothelial growth factor receptor 2 (VEGFR2) and sensitivity to VEGF-A (discussed by Silva et al. 2008) suggesting that these integrins are not required for blood vessel formation but regulate their rate of growth.

Integrin αvβ8-knock out mice demonstrate severe vascular defects resulting in haemorrhaging, predominantly in the central nervous system, with a large proportion of mice dying shortly after birth and some earlier (Zhu et al. 2002). Knockout of β8 in Muller glia or retinal ganglion cells promotes vascular abnormalities that match retinal TGFβ1-knockout, suggesting αvβ8 is necessary for TGFβ1–dependent retinal maturation (Arnold et al. 2012). Integrin αvβ8 is not expressed on endothelial cells but is expressed on neural peri-vascular astrocytic cells where it binds and activates latent TGFβ1, which promotes increased stability and differentiation of blood vessels (Cambier et al. 2005).

Analysis of αvβ8 function was examined in astrocytoma by using orthotopic transgenic cell lines. Intra-cranial injection of human β8-low astrocytoma cells (U87) resulted in haemorrhagic tumours, whereas β8-positive cells formed microscopic tumours with well-defined vasculature. Orthotopic injection again showed a well-established vasculature in β8 wild-type transformed astrocytes and a disorganised haemorrhagic vasculature in the absence of β8. Moreover, endothelial cells examined from β8^−/−^ mice express approximately two- to three-fold less phospho-Smad 2 and 3 suggesting that less active TGFβ is present in β8-null mice. However, no significant difference was seen in tumour volume in β8^−/−^ and wild-type mice, although the former exhibited reduced survival rates because of haemorrhage (Tchaicha et al. 2010). In separate studies, the over expression of β8 in U87 was shown to result in brain tumours with organised non-haemorrhagic vasculature and this affect required the activation of latent TGFβ1 (Tchaicha et al. 2011).

A key mediator between intracellular TGFβ signalling and transmembrane integrins in endothelial cells is endoglin, a TGFβ superfamily accessory receptor that is expressed in the angiogenic vessels of brain, lung, breast, stomach and colon cancers (Minhajat et al. 2006). In response to murine embryonic endothelial cells binding fibronectin via integrin α5β1, TGF-β1-induced Smad1/5/8 phosphorylation increases in an endoglin-dependent manner. Reciprocally, endoglin is necessary for TGFβ1-induced phosphorylation of β1 integrin threonines 788/789 and FAK, whereas fibronectin/endoglin cross-talk maintains endothelial cell survival (Tian et al. 2012). Perhaps unsurprisingly, endoglin is a prognostic marker in colorectal cancers with correlation to lymph node and liver metastases (Saad et al. 2004).

5) **TGFβ regulates inflammatory and immune cells to modulate cancer progression**

When integrin-LAP binding is prevented by an RGD to RGE mutation in LAPβ1, mice exhibit a multi-organ inflammatory response that mirrors global TGFβ1-knockout (Yang et al. 2007). Since the effective absence of both αvβ6 and αvβ8 also results in multi-organ inflammation, recapitulating global TGFβ1 deficiency, this suggests that none of the other αv integrins are required to regulate these particular TGFβ1-dependent effects (Aluwihare et al. 2009). However, these studies have only assessed the integrin-dependent activation of latent TGFβ during development and birth but not in adult mice or in pathological situations such as cancer. Thus, the possibility exists that the activation of TGFβ by any αv integrin can influence local inflammatory and immune cells in adult animals. This is important as TGFβ1 promotes immunosuppressive effects on various effector T-cells (for a review, see Travis and Sheppard 2014) and induces tumour-promoting phenotypes in both neutrophils and macrophages.

Travis et al. (2007) reported that the loss of αvβ8 on dendritic cells results in inflammatory bowel disease (Travis et al. 2007). The effect is attributable to an inability of αvβ8-deficient dendritic cells to activate TGFβ locally and thus to promote the differentiation of naïve T-cells into TReg via Foxp3 transcription factor (Chen et al. 2003). TReg cells are a subpopulation of CD4^+^ CD25^+^ T cells whose functions encompass the immunosuppression of effector T-cells including the maturation of CD8 cytotoxic T cells (McNally et al. 2011) and maintaining immunological tolerance (discussed in Travis and Sheppard 2014). Moreover, these differentiated cells suppress their production of helper T cell cytokines, instead secreting TGFβ1, further amplifying TGFβ1 signalling and its immunosuppressive effects (Chen et al. 2003). These data correlate with the observation that high levels of TRegs in cancer tissues are associated with poor overall survival from several types of solid cancer (Shang et al. 2015). TReg accumulation correlates with tumour progression in breast, ovarian and pancreatic cancer, although the effect of TRegs on prognosis depends on factors such as tumour location and inflammatory status (Oleinika et al. 2013; Schmidt et al. 2016). Although, to date, no pre-clinical studies have made a detailed examination of immune infiltrates in response to blockade of αv integrins, carcinomas expressing αvβ6, astrocytomas expressing αvβ8 and desmoplastic tumour myofibroblasts expressing αvβ5 and αvβ1, are likely to regulate TReg levels in the TME.

TGFβ1 also promotes the formation of tumour-promoting M2 tumour-associated macrophages and N2 tumour-associated neutrophils (Gong et al. 2012; Fridlender et al. 2009). Macrophages present as having “M1” anti-tumour or “M2” tumour-promoting characteristics. Macrophages co-cultured with ovarian carcinoma cells significantly increase the transcription of TGFβ1, TGFBRI and TGFBRII (Hagemann et al. 2006). TGFβ1 stimulates monocyte recruitment and alters the inflammatory gene expression profile of macrophages by increasing metastasis-associated interleukin-6 (IL-6) and suppressing cytokines such as IL-10 and chemoattractants CCL3 and CCL4 (Krstic and Santibanez 2014). TGFβ1 stimulation of macrophages also promotes angiogenesis under hypoxic conditions by the elevated production of VEGF, MMP-9 and VEGF receptor Flk-1 expression (Jeon et al. 2007). High levels of M2 macrophages correlate with poor survival from cancer including pancreatic and cervical cancer (Petrillo et al. 2015; Hu et al. 2016), gastric cancer spread (Yamaguchi et al. 2015) and relapse after chemotherapy (Hughes et al. 2015). Indeed, pre-clinical studies suggest M2 cells can promote metastasis (Ding et al. 2015; Wu et al. 2015; Yang et al. 2015).

Similarly, tumour-associated neutrophils polarised to the N2 state are also pro-tumourigenic as reviewed recently (Sionov et al. 2015). Thus, depletion of N2 neutrophils within TGFβ1-rich mouse tumours significantly reduces tumour size, supporting the concept that separate populations of anti-tumour and pro-tumour neutrophils are regulated by active TGFβ (Fridlender et al. 2009). Recent studies by Sagiv et al. (2015) suggested neutrophils can be segregated according to their cell density, whereby low-density neutrophils are tumour-promoting and high-density neutrophils are tumour-suppressive. The authors discovered that tumour progression in mice correlated with an increase in the fraction of circulating low-density neutrophils, eventually becoming the dominant fraction; in tumour-free mice, 95 % of circulating neutrophils had the high-density phenotype. Moreover, the authors discovered that TGFβ1 was required and sufficient to promote the transition of the high-density neutrophils into the tumour-supportive low-density neutrophils. Functionally, the high-density neutrophils suppressed tumour growth and were cytotoxic in vitro towards tumour cells. In contrast, the low-density neutrophils were deficient in cytotoxic H_2_O_2_, exhibited reduced chemotaxis towards the tumour and were less phagocytic. In addition, low-density neutrophils produced less inflammatory cytokines and were able to suppress the proliferation of CD8+ cytotoxic T-cells and thus effectively promoted tumour growth.

Overall, TGFβ has tumour-promoting and -immunosuppressive effects on several immune cell types in the tumour stroma. However, more studies are needed to determine what proportion of TGFβ activation of immune cells in the tumour stroma is solely integrin-dependent, so that therapeutic targeting of relevant integrins can suppress the pathogenic effects of active TGFβ on tumour-promoting immune components.

## Future of drug targeting integrin-dependent activation of TGFβ in cancer

In this review, we discussed the indirect effects on tumour progression exerted by αv integrins because of their ability to activate latent TGFβ1. However, all αv integrins can signal via their cytoplasmic tails and some (e.g., αvβ3 and αvβ6) can generate pro-tumourigenic signals including those for growth, survival and the secretion of proteases, factors that can enhance tumour progression. Thus, the fraction of an integrin’s ability to promote cancer via its cytoplasmic tail versus its ability to activate TGFβ is unclear. Regardless, the direct inhibition of the ligand-binding site of αv integrins is sufficient to block their capacity to activate TGFβ1 and to generate survival signals and has been used in pre-clinical and clinical studies (Eberlein et al. 2013; Marsh et al. 2008).

Of the integrins that can potentially activate TGFβ1, only αvβ3-specific antibodies and peptides have been used in humans as part of anti-cancer therapies (Posey et al. 2001; Hersey et al. 2010; Khasraw et al. 2016) but none has achieved significant clinical success. Whereas some cancers express high levels of αvβ3 (e.g., melanoma and glioblastoma), the discovery that αvβ3 negatively regulates the endothelial cell growth factor receptor VEGFR2 means that blockade of αvβ3 will result in increased angiogenesis and tumour growth (see above). However, Reynolds et al. (2009) reported that sub-therapeutic concentrations of Cilengitide, an αvβ3/αvβ5 peptide inhibitor, enhanced angiogenesis and, as new blood vessels tend to be leakier, Wong et al. (2015) used this to show that pre-dosing with Cilengitide improved drug accessibility to a variety of experimental tumours. Thus, targeting αvβ3 may still be therapeutic but possibly not by inhibiting TGFβ1 activation. Recently, Merck published results of the POSEIDON trial. Their pan-αv blocking humanised antibody Abituzumab was compared with the standard of care (cetuximab, an EGFR inhibitor and irinotecan, a topoisomerase 1 inhibitor) in metastatic colorectal cancer (CRC; Elez et al. 2015). Data from the whole cohort showed that combining Abituzumab with cetuximab and irinotecan provided no significant survival benefit versus the cetuximab and irinotecan combination alone. However, when the expression levels of the different αv inetrgins on the tumour tissue were assessed, the data showed that the risk of death for patients with high αvβ6 (above the median histological score) was reduced by 59 % by the addition of Abituzumab to the treatment regimen. Overall, the data suggest that targeting αvβ6 with inhibitory antibodies could be positively therapeutic for patients whose cancer over-expresses αvβ6. It is worth recalling that, in those studies with large enough patient cohorts, high αvβ6 correlated with poor survival and, thus, the identification of those patients by the simple immunohistochemistry of biopsies might be sufficient to select only the likely responders to αvβ6-blocking therapy.

Antibody targeting of integrin αvβ6 in humans is work in progress. Thus, Biogen-Idec have developed the humanised αvβ6-blocking monoclonal antibody STX-100, which is being used in idiopathic fibrosis studies (clinicaltrials.gov# NCT01371305). AstraZeneca-Medimmune have developed the entirely human monoclonal antibody 264RAD, which blocks αvβ6 but also has some blocking activity of αvβ8 (Eberlein et al. 2013). To date, this antibody has not entered clinical trials but speculation regarding its potential effects is of interest, given the results of the POSEIDON study, which has suggested that the blockade of αvβ6 is likely to be therapeutic in colon cancer (Elez et al. 2015). Thus, Eberlein and colleagues (2013) reported the complete suppression of the growth of xenografts of the oral squamous carcinoma cell line Detroit-562 at doses of 5 and 20 mg/kg 264RAD and this correlates with the loss of expression of the target integrin αvβ6 on the residual tumour. Moore and colleagues (2014) also noted that 264RAD-induced breast tumour xenograft suppression also correlates with loss of αvβ6 expression. Since antibody 264RAD operates as a ligand mimetic, we consider it worth noting that, in a recent study, GSK also reported, from in vitro studies, that the exposure of cells to LAP or A20FMDV2, a high-affinity αvβ6-specific 20-amino-acid peptide derived from foot-and-mouth-disease virus, causes a rapid ligand-induced internalisation of αvβ6 but a slower re-expression (Slack et al. 2016). Thus, perhaps ligand-induced transient loss of cell-surface αv integrins may be a goal of future anti-cancer therapeutics, since this might be more efficient as a means of suppressing local TGFβ1 activation than attempting to achieve high enough concentrations of an extracellular inhibitor that can completely suppress the binding of the integrin to LAP.

Antibody blockade of αvβ6 may not be the only therapeutic strategy for regulating TGFβ1 activity. Merck has generated small-molecule inhibitors of αvβ6, αvβ5 and αvβ3 (Goodman et al. 2002) and GSK have recently produced naphtyridine-derivative small molecule antagonists of αvβ6 (Patent WO 2014154725 A1). Whereas neither company has used their compounds clinically, if these small molecules can also operate as ligand-mimetics, they might compare favourably with inhibitory antibodies that are likely to have better pharmacokinetics.

We should also mention the potential added value of the therapeutic targeting of integrins as a means of regulating TGFβ1 activity in cancer and fibrosis as opposed to directly targeting the TGFβ1 signalling pathway. Thus, the blockade of TGFβ receptors with small-molecule kinase inhibitors or of TGFβ itself with inhibitory antibodies would promote a whole body inhibition of TGFβ activity, whereas integrin blockade produces local control of TGFβ activation. Since, as mentioned above, TGFβ is required for normal tissue regulation, integrin targeting would minimise off-target effects.

## Concluding remarks

**Fig. 2:**
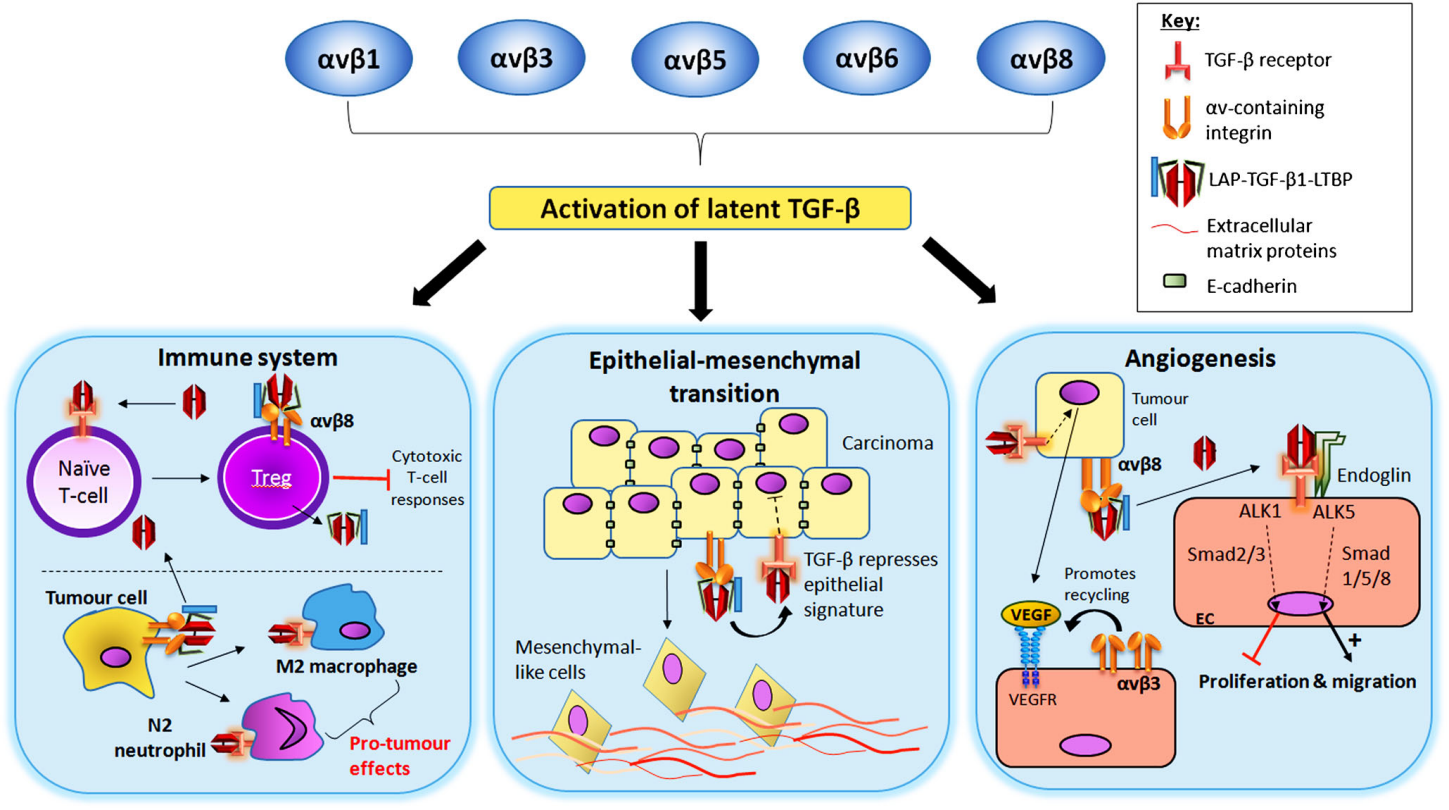
Contribution of integrin-mediated TGFβ1 activation of pro-tumour mechanisms via regulation of cells of the immune system, promotion of epithelial-mesenchymal transition and angiogenesis. *Immune system* 1) Active TGFβ stimulates naive T-cell-to-Treg differentiation (*top*). Tregs exhibit αvβ8-mediated TGFβ1 activation, amplifying the differentiation of other naive T-cells. Tregs suppress anti-tumour T-cell responses. Tumour cell integrins mediate TGFβ1 activation (*bottom*). Active TGFβ1 stimulates macrophage and neutrophil pro-tumour responses. *Epithelial-to-mesenchymal transition* TGFβ1 stimulation of epithelial cells represses epithelial gene signatures, e.g., cell-cell adhesion molecule E-cadherin (*top*). This promotes a more mesenchymal invasive cell phenotype (*bottom*). *Angiogenesis* TGFβ1 stimulation of tumour cells increases VEGF expression, which binds to its receptor on local endothelial cells. Integrin αvβ3 promotes activation and recycling of VEGFR (*left*). The αvβ8-mediated activation of TGFβ1 facilitates TGFβ activation of local endothelial cells, which can promote or inhibit angiogenesis via separate TGFβ receptors (*right*)

Integrin activation of latent TGFβ1 can be a significant component of the way that tumours develop and respond to their stroma. First, the ability of αvβ6 and αvβ8 integrins to activate TGFβ1 was established as a major platform for the normal homeostatic regulation of TGFβ1 activity. Second, the discovery that both αvβ5 and αvβ1 on fibroblasts also activated latent TGFβ1 in a biomechanical way (like αvβ6) indicated that stromal cells could also contribute to local TGFβ1 activity. Finally, the additional revelation that increasing stromal stiffness reduced the activation threshold for the force required to activate TGFβ1 introduced the likelihood that pathological stromal fibrosis was driven by an amplification of activation of abundant latent TGFβ1 in the matrix generated by multiple αv integrins. However, although multiple mechanisms exist by which TGFβ1 modulates the tumour microenvironment to promote cancer (Fig. 2; Pickup et al. 2013), we still have a relative paucity of empirical in vivo data firmly establishing those TGFβ1-driven stromal responses that are the direct result of the αv integrin activation of TGFβ. We clearly lack certain reagents and model systems that would allow us fully to characterise those αv integrins that activate TGFβ at various times and stages in cancer progression. Thus, we lack certain tissue-specific inducible αvβ integrin knockout mice (including *itgb6*) that would permit researchers, over the course of the development of a cancer, temporally to eliminate an αvβ integrin and examine its contribution to TGFβ1-driven tumourigenesis. Subsequently, by introducing therapeutics to these same integrins, we could establish when the most effective therapeutic window occurs and also whether cancers reach a point at which the targeting of integrin-dependent TGFβ1 activation is no longer effective, perhaps because so many processes (multiple integrins, tissue stiffness, proteases) are simultaneously able to activate the latent TGFβ. We have mentioned the absence of antibodies that would allow us to determine the expression of αvβ1 and αvβ8 in tissues and, similarly, no αvβ8 blocking antibodies are available for pre-clinical studies. Therefore, as these reagents become available, we may better be able to delineate an appropriate clinical strategy by the study of the temporal increase in the number of diverse mechanisms for latent TGFβ activation that accumulate during cancer development. However, although we may lack some data that will refine our treatments, αv integrins must now be considered as *bona fide* targets for anti-TGFβ therapy in fibrosis and cancer.
